# Propensity-Matched Survival Analysis of Upper Urinary Tract Urothelial Carcinomas between End-Stage Renal Disease with and without Kidney Transplantation

**DOI:** 10.1155/2019/2979142

**Published:** 2019-04-01

**Authors:** Hao Lun Luo, Po Hui Chiang, Yuan Tso Cheng, Yen Ta Chen

**Affiliations:** ^1^Department of Urology, Kaohsiung Chang Gung Memorial Hospital and Chang Gung University College of Medicine, Kaohsiung, Taiwan; ^2^Graduate Institute of Medicine, College of Medicine, Kaohsiung Medical University, 83301 Kaohsiung, Taiwan

## Abstract

Urothelial carcinoma is the most common cancer following kidney transplantation (KT) in Taiwan. Unusual presentation of upper urinary tract urothelial carcinoma (UTUC) is noted in Taiwan and China. As the post-KT-UTUC oncological course is not fully understood, the aim of this study is to identify postulated significant differences for the clinical cancer course of UTUC among end-stage renal disease (ESRD) patients with and without KT. From 2005 January to 2016 March, 194 ESRD patients underwent radical nephroureterectomy due to UTUC in our hospital. The parameters were obtained from the chart record and pathology report. SPSS version 21 software was used for all statistical analyses. Unequal matching created study groups wherein a 0.2 caliper width was performed for adjusting these confounding pathological factors. Propensity score-matching cohort was performed for each population first, and then for all the study patients. We observed that the average age of UTUC in ESRD patients after KT was younger than in those without KT. The pathological factors such as stage, bladder cancer history, papillary structure, lymphovascular invasion, and variant histology were equal in these two groups. However, younger onset (p<0.001), more multifocal tumors, and carcinomas in situ were observed in post-KT UTUC (p<0.001 and 0.006, respectively). After adjustment of pathological factors by propensity score-matched analysis, the 5-year systemic UTUC recurrence was significantly more in ESRD after KT compared with ESRD without KT (p=0.03). No obvious difference in 5-year cancer related death could be observed between these two groups (p=0.314). Post-kidney transplantation upper urinary tract urothelial carcinoma in Taiwan is relatively common, has younger onset, and is associated with aggressive pathological features. The oncologic outcome of UTUC after KT is poor in our observation, even after propensity scored-matched analysis. It indicates the immunosuppression status is still associated with more malignant UTUC behavior.

## 1. Introduction

Urothelial carcinoma is the most common cancer following kidney transplantation (KT) in Taiwan [1]. A Taiwan nationwide population-based cohort revealed chronic kidney disease is commonly associated with upper urinary tract urothelial carcinoma (UTUC) [2]; therefore, post-KT UTUC screening is an important issue for post-KT patient care. A recent systemic review concerning urologic tumor recurrence risk after kidney transplantation revealed that KT does not affect the oncologic outcome for low-risk renal cell carcinoma and prostate cancer [3]. However, unusual presentation of UTUC is noted in both Taiwan and China [4, 5]. The post-KT UTUC oncologic course is not fully understood; therefore, the aim of this study was to identify the clinical cancer course of UTUC among end-stage renal disease (ESRD) patients with and without KT by propensity-matching analysis.

## 2. Material and Methods

From 2005 January to 2016 March, 172 ESRD patients underwent radical nephroureterectomy due to UTUC. All patients underwent preoperative computed tomography (CT) and cystoscopy to determine if they had concurrent distant metastasis or urinary bladder urothelial carcinoma. Postoperative CT and cystoscopy were performed regularly to detect any disease recurrence. The definition of local recurrence and distant metastasis was disease relapse over and distant to any previous retroperitoneal surgical area. Residual urinary tract system recurrences were not included as endpoints. This study was approved by our Institution Review Board, IRB number 201600750B0.

The clinical parameters and pathological features were obtained from our chart and pathology report. SPSS version 21 software was used for all statistical analyses. Chi-square tests and 2-sample* t*-tests were used for intergroup comparisons, and the Kaplan-Meier test was used for time-to-event analysis. Using NCSS software, (NCSS 10; NCSS statistical software, Kaysville, Utah), the optimal method was used to create unequally-matched study groups with a 0.2 caliper width. After adjusting for these confounding pathological factors, binary logistic regression was used to evaluate the interventional factor of KT. Propensity score-matching cohort was performed for each population first and then for all the study patients. A p value of ≤0.05 was defined to be statistically significant.

## 3. Results

**Figure 1:**
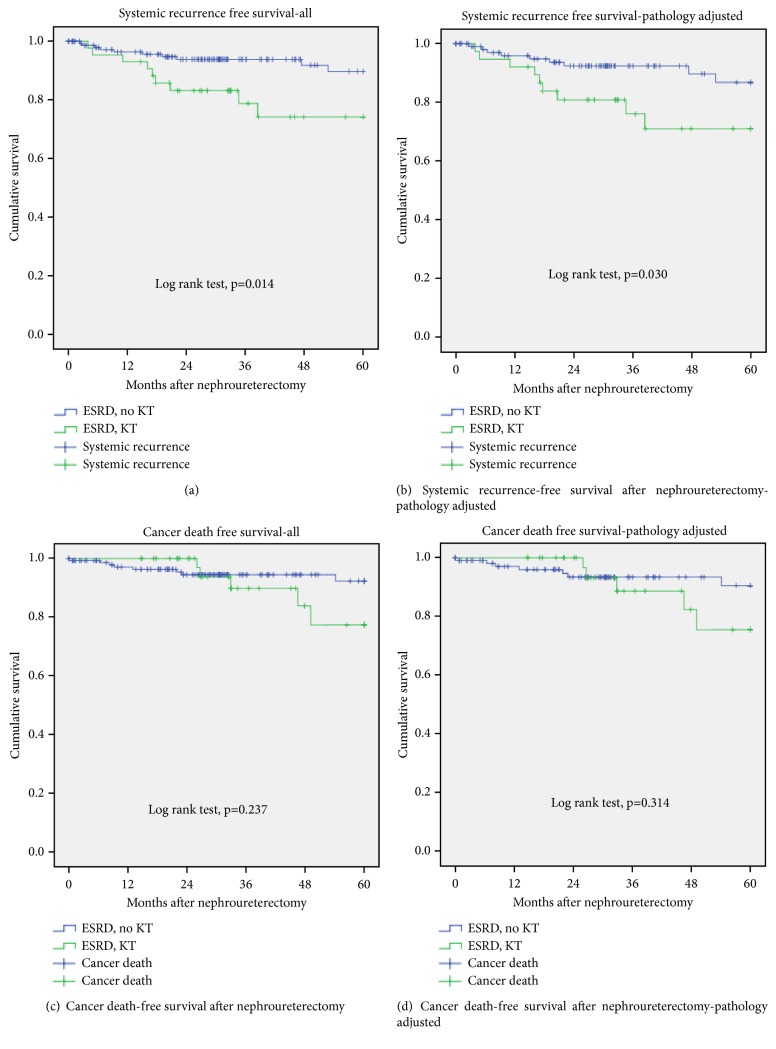


Table 1 reveals that the average age of UTUC in ESRD patients after KT was younger than in those without KT. The pathological factors such as stage, bladder cancer history, papillary structure, lymphovascular invasion, and variant histology were equal in these two groups. However, younger onset (p<0.001), more multifocal tumors, and carcinomas in situ were observed in post-KT UTUC (p<0.001 and 0.006, respectively). In order to decrease the bias from pathological factors, we performed propensity score-matching pair analysis for pathological features in both groups. The adjusted cohort still revealed younger age in patients with ESRD after KT (p=0.001). After adjustment of pathological factors by propensity score-matched analysis, the 5-year systemic UTUC recurrence was significantly more in ESRD after KT compared with ESRD without KT (p=0.03). No obvious difference in 5-year cancer-related death could be observed between these two groups (p=0.314). The Kaplan Meier plot is shown in Figure 1, which reveals significantly increasing systemic recurrence and borderline increase of cancer-specific mortality in patients with ESRD after KT (primary and pathology-adjusted study group).

## 4. Discussion

In Taiwan and China, unusually high prevalence and female predominance of upper urinary tract urothelial carcinoma has been noted [4, 5]. In recent reports, relatively high prevalence of UTUC with aristolochic acid (AA) mutational signatures was found in Taiwan [6, 7]. Aristolochic acid-related UTUC is associated with a specific oncologic pattern [8], namely, aristolochic acid nephropathy, which is a toxic interstitial nephropathy caused by ingestion of plants containing aristolochic acids (AA) as part of traditional phytotherapies (formerly known as Chinese herb nephropathy) [9]. There is relatively high prevalence of UTUC and subsequent bladder cancer for such patients with AA-related nephropathy after KT [10]. Therefore, urinary bladder or upper urinary tract urothelial carcinoma is the leading malignancy after KT in Taiwan, and standardized incidence ratios both are more than 40 [1].

The primary and pathology-adjusted cohort revealed significantly younger onset of UTUC in the post-KT group. The common risk factors for post-transplantation malignancy are immunosuppression and oncogenic viral infections [11]. We previously reported that post-KT patients were at risk of human polyomavirus infection and that this is associated with earlier onset of UTUC in Taiwan [12]. Besides, we also observed there were more aggressive pathological features such as carcinoma in situ and multifocality in post-KT UTUC patients compared with controlled-ESRD patients. The urothelium cancerization in post-KT UTUC patients needs further investigation. According to this study, UTUC screening should be started earlier due to relatively younger onset in post-KT patients. There was no urine efflux from native kidney due to end-stage renal disease, and urine cytology could only detect the urinary bladder lesion; therefore, we suggest that clinical physicians should perform kidney ultrasound and even abdominal computed tomography for any suspicious lesion detected, particularly in Taiwan, a high UTUC-prevalent area. Earlier detection of UTUC might improve the oncological outcome of post-KT UTUC.

In this study, we include UTUC patients with baseline ESRD in southern Taiwan. A previous study revealed high incidence of residual urinary tract urothelial carcinoma recurrence and suggested more intensive urinary tract follow-up protocol [10]; however, the local regional lymph node recurrence or distant metastasis outside the residual urinary tract is the major cause of cancer-specific mortality and should be carefully monitored by image study. Though the oncologic outcome of low-risk RCC and prostate cancer after KT is similar [3], the evidence concerning oncologic outcome of UTUC after KT is difficult to accumulate owing to its relatively rare incidence. We have previously reported preliminary data about the characteristics of post-KT UTUC revealing that the UTUC after KT has younger onset and more aggressive pathological features; however, bias from unequal pathological features might present unsuitable outcome analysis [13]. Post-KT immunosuppression status is a well-known risk factor for cancer development [11] and whether the post-KT effect will cause worse cancer outcome or not should be carefully evaluated. In this study, we balanced the bias from baseline pathological features by propensity score-matched analysis and identified that the patients with UTUC after KT had higher risk of systemic disease recurrence if they underwent standard nephroureterectomy alone. More intensive treatment protocols such as perioperative systemic therapy or extensive lymph node dissection might improve oncologic outcome of post-KT UTUC [14, 15]. We did not observe statistical differences for cancer-specific death between these two groups. This might be attributed to the relatively shorter survival time in ESRD patients without KT to observe the endpoint, and ESRD patients with KT might be more eligible for intensive cancer treatment thereby prolonging survival outcome.

The limitation of this study is its retrospective study design and asymmetric clinical parameters. Significantly younger age in post-KT UTUC could not be adjusted because UTUC is a disease commonly occurring in the elderly, but the post-KT UTUC patients were much younger. We could only use the propensity score-matched method to compare these two groups without equally matched pathological confounding factors as best as possible. Post-KT UTUC is a rare condition, but the incidence is relatively high in Taiwan, and therefore we can accumulate sufficient patient numbers to analyze the relationship between post-KT status and UTUC cancer behavior. Further detailed information such as somatic mutation of tumors or oncogenic virus status might be helpful to understand the early carcinogenesis and aggressive cancer behavior after KT.

## 5. Conclusion

Post-kidney transplantation upper urinary tract urothelial carcinoma (post-KT UTUC) in Taiwan is relatively common, has younger onset, and is associated with aggressive pathological features. Consequently, the oncologic outcome of UTUC after KT is poor in our observation, with the oncologic outcome being still worse in patients with KT than without KT even after adjustment of the pathological factors. This indicates the immunosuppression status is still associated with more malignant UTUC behavior.

## Figures and Tables

**Table 1:** Patient demography.

	Primary cohort			Propensity-matched cohort		
	ESRD	ESRD	*p* value	ESRD	ESRD	*p* value
	no KT	KT		no KT	KT	
	n=151	n=43		n=110	n=38	
FU (months)	43.7±35.4	50.2±35.8	0.289	43.2±35.8	50.8±36.6	0.265
Age(years)	63.4±11.1	56.7±7.4	*<0.001*	63.7±11.1	57.4±7.1	*0.001*
Male/Female	95/56	28/15	0.791	41/69	14/24	0.962
pT stage			0.227			0.831
pTa/Tis	48	13		37	10	
pT1	49	10		32	10	
pT2	22	9		16	8	
pT3	28	10		22	9	
pT4	4	1		3	1	
Papillary	123	33	0.492	91	29	0.384
Multifocal^*∗*^	56	26	*<0.001*	51	21	0.344
BC history	79	19	0.347			
High grade	139	41	0.461	101	36	0.554
LVI	21	6	0.994	14	6	0.634
CIS	73	31	*0.006*	65	26	0.308
Variant UC	43	11	0.709	33	10	0.666
5-year SR	10	9	*0.014*	9	9	*0.030*
5-year CD	8	5	0.237	7	5	0.314

FU: follow-up duration, BC: bladder cancer, LVI: lymphovascular invasion, CIS: carcinoma in situ, UC: urothelial carcinoma, SR: systemic recurrence, and CD: cancer-related death.

^*∗*^Multifocal: concurrent renal pelvis and ureteral cancer.

## Data Availability

The data used to support the findings of this study are available from the corresponding author upon request.
